# The dynamics of extracellular DNA associates with treatment response in patients with rheumatoid arthritis

**DOI:** 10.1038/s41598-022-23954-8

**Published:** 2022-12-06

**Authors:** Kristína Macáková, Júlia Illésová, Vanda Mlynáriková, Alexandra Lesayová, Barbora Konečná, Barbora Vlková, Peter Celec, Emöke Šteňová

**Affiliations:** 1 Institute of Molecular Biomedicine, Faculty of Medicine, Comenius University, Bratislava, 81108 Slovakia; 2 National Institute of Rheumatic Diseases, Piešťany, 92101 Slovakia; 3 Edinburgh Medical School: Biomedical Sciences, University of Edinburgh, Edinburgh, EH8 9XD UK; 4 Institute of Pathophysiology, Faculty of Medicine, Comenius University, Bratislava, 81108 Slovakia; 5 Institute of Molecular Biology, Faculty of Natural Sciences, Comenius University, Bratislava, 84215 Slovakia; 6 1st Department of Internal Medicine, Faculty of Medicine, University Hospital, Comenius University, Bratislava, 82101 Slovakia

**Keywords:** Rheumatic diseases, Biomarkers

## Abstract

Rheumatoid arthritis (RA) as a chronic autoimmune inflammatory disease increases extracellular DNA (ecDNA). Our previous study has shown that anti-inflammatory treatment reduces ecDNA, but it is unclear whether there is an association with treatment response. The aim of this study was to analyze the changes of ecDNA induced by biological disease-modifying antirheumatic drugs (bDMARDs) in RA patients with an emphasis on the subcellular origin of ecDNA. Plasma samples from 40 RA patients were collected in three different time-points: before treatment with bDMARDs as well as 3 and 12 months following treatment initiation. Total, nuclear and mitochondrial ecDNA was quantified using fluorometry and real-time PCR. Disease activity score (DAS28) and C-reactive protein (CRP) were used to monitor the clinical status and the response to treatment. Treatment with bDMARDs elicited an overall improvement of the clinical status: DAS28 and CRP showed a significant decrease by 54% and 43%, respectively, after 3 months of treatment. A significant decrease of total ecDNA by 60% and nuclear ecDNA by 58% was detected only in good responders after 3 months of bDMARDs treatment. No significant changes of plasma ecDNA concentration were observed in moderate and non-responders. Deoxyribonuclease activity was not affected by the treatment. None of the analyzed biomarkers differed between the groups at baseline. Plasma ecDNA especially of nuclear origin could potentially be useful to monitor the treatment response in RA. Further studies should shed light on disease-treatment interplay implicated in ecDNA origin potentially linked to neutrophil extracellular traps.

## Introduction

Rheumatoid arthritis (RA) is a chronic autoimmune inflammatory disease predominantly targeting joints. Although the RA prevalence is around 1%, its etiopathogenesis has not yet been sufficiently described^1^. As such, the treatment is mainly symptomatic and therefore does not cure the disease cause. The traditional way of treating RA patients involves non-steroidal anti-inflammatory drugs, glucocorticoids, and disease-modifying antirheumatic drugs (DMARDs)^2^, The available RA therapy made great progress with patients achieving remission shortly after the treatment initiation, especially after introduction of biological disease-modifying antirheumatic drugs (bDMARDs)—monoclonal antibodies and decoys targeting soluble extracellular and cell membrane-associated proteins involved in induction and persistence of inflammation and, thus, key factors in the pathogenesis of RA^1,3^. Despite great therapeutic progress, a group of patients seems to be unresponsive to some of the bDMARDs. There is a need for biomarkers for early diagnosis as well as for stratification of patients’ response to treatment. Inflammatory and autoimmune diseases are associated with higher concentrations of extracellular DNA (ecDNA)^4,5^. EcDNA is, however, more than just a biomarker of tissue damage or inflammation as it stimulates immune cells depending on the ecDNA origin^6,7^. Nuclear DNA (ncDNA) and mitochondrial DNA (mtDNA) can be distinguished based on the subcellular origin; with mtDNA being more immunogenic due to its prokaryotic origin^8^. Increased concentrations of ecDNA observed in RA patients, and the associations with disease progression suggest that ecDNA might be a potential marker for monitoring of the clinical status as well as for the prognosis of treatment efficacy in RA. Importantly, this important and clinically relevant topic has been analyzed in only few studies^9–12^. In the study of Rykova et al., dynamics of subcellular origin of ecDNA was examined. Results showed that the application of bDMARDs had no impact on ecDNA concentration, while the disease activity after bDMARDs application decreased^10^. In our previous study, we demonstrated that the administration of bDMARDs decreases ecDNA and mtDNA after six months of treatment^12^. Differences in the dynamics of ecDNA related to the RA treatment were also observed in the study of Hashimoto et al. where good responders had higher ecDNA concentrations four weeks after the bDMARDs treatment initiation^11^. Discrepancies in the observed ecDNA concentrations can be caused by different methods applied, but also by the different timepoints selected for the quantification. A long-term observation would, thus, be ideal. This was the motivation for an extension of the observation period and quantification of ecDNA twelve months after treatment initiation in the current study.

Various processes can cause the release of DNA from cells. One source of ecDNA are neutrophil extracellular traps (NETs), structures produced in specific conditions as a response to the presence of infectious or sterile stimuli^13–15^. Besides ecDNA, NETs contain proteins such as histones, antimicrobial peptides and enzymes—peptidyl-arginine deiminase (PAD), neutrophil elastase and myeloperoxidase^13,16,17^. The activity of PAD enzymes leads to citrullination of proteins, which is one of the main triggers for formation of specific antibodies and production of proinflammatory cytokines—typical for several autoimmune disorders^13,18^. NETs production in RA can also lead to immune cell infiltration of the joints and cartilage degradation^19^. The released ecDNA interacts with Toll-like receptors and activates inflammatory pathways^20^. It was also described that ecDNA binds to protein missagregates, which might underlie neurodegenerative diseases such as Alzheimer’s disease^21,22^.

EcDNA is present in plasma under physiological conditions, however, mostly at low concentrations ensured by lytic activity of deoxyribonucleases (DNases)^9–12,20–24^. As such, DNase regulates ecDNA concentration; activity of this enzyme hence plays a crucial physiological role in prevention of auto-immunity^24^. Reduced DNase I activity contributes to the development of systemic lupus erythematosus, but its role in other autoimmune diseases including RA is not clear^25,26^.

This study is a follow up to our first investigation regarding the dynamics of ecDNA in RA in which patients with no response were not included and the period of observation was extended from six to twelve months. The aim of this study was to analyze the dynamics of plasma ecDNA and its subcellular origin dynamics as well as DNase activity upon bDMARDs treatment in relation to treatment response.

## Results

### Clinical parameters

**Figure 1:**
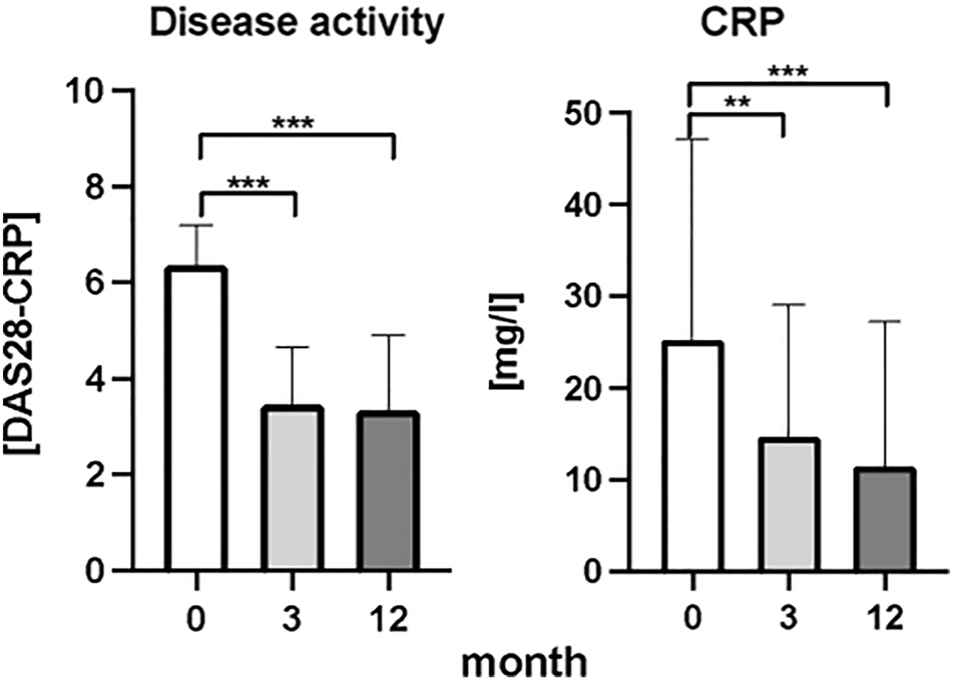
Clinical status of patients with rheumatoid arthritis: disease activity score (DAS28) and C-reactive protein (CRP), before the bDMARDs treatment initiation and 3 and 12 months during ongoing treatment (*p* < 0.05 = *, *p* < 0.01 = **, *p* < 0.001 = ***).

**Figure 2:**
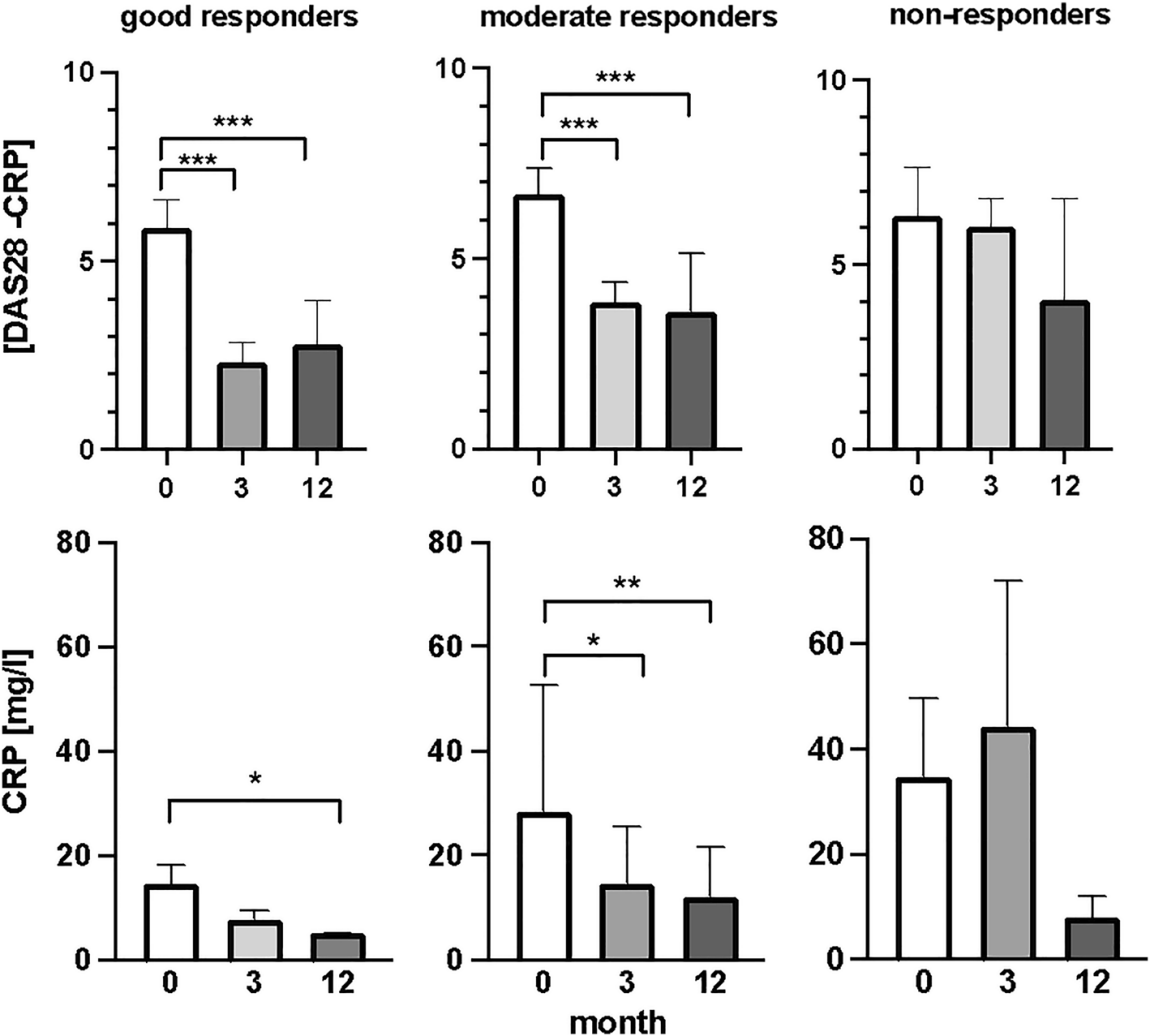
Clinical status of patients with rheumatoid arthritis divided into groups based on their response to biological treatment (good, moderate, and non-responders). Before initiation of bDMARDs and 3 and 12 months during ongoing treatment (*p* < 0.05 = *, *p* < 0.01 = **, *p* < 0.001 = ***).

When all patients were analyzed with clinical parameters disease activity score evaluating 28 joints (DAS28) and C-reactive protein (CRP), a significant decrease was observed after the first three months of bDMARDs treatment Fig. 1. Levels of DAS28 have halved after the first three months of biological treatment (Fig. 1), (*p* < 0.001, F = 134.9), while concentration of CRP in plasma of RA patients during the same time decreased by 43%. From the third to the twelve months after the bDMARDs no significant decrease of both parameters were detected. According to the responsivity to the treatment, good responders showed a significant decrease in DAS28 score at three months of treatment (Fig. 2), (*p* < 0.001, F = 106.1). DAS28 decreased by 61% after three months. In the period of the time from the third to twelve months of bDMARDs therapy no significant decrease was observed. Regarding the CRP parameter, a decrease by 50% was observed only in the period from the baseline and twelve months after the treatment (Fig. 2), (*p* < 0.05, F = 12.25). DAS28 in moderate responders showed significant decrease by 42% (Fig. 2), (*p* < 0.001, F = 84.09). No significant changes occurred from the third to twelve months of the treatment. In moderate responders, CRP significantly decreased by 49% again only after the first three months of the treatment (Fig. 2), (*p* < 0.05, F = 12.11).

### Concentration of ecDNA and the subcellular origin

**Figure 3:**
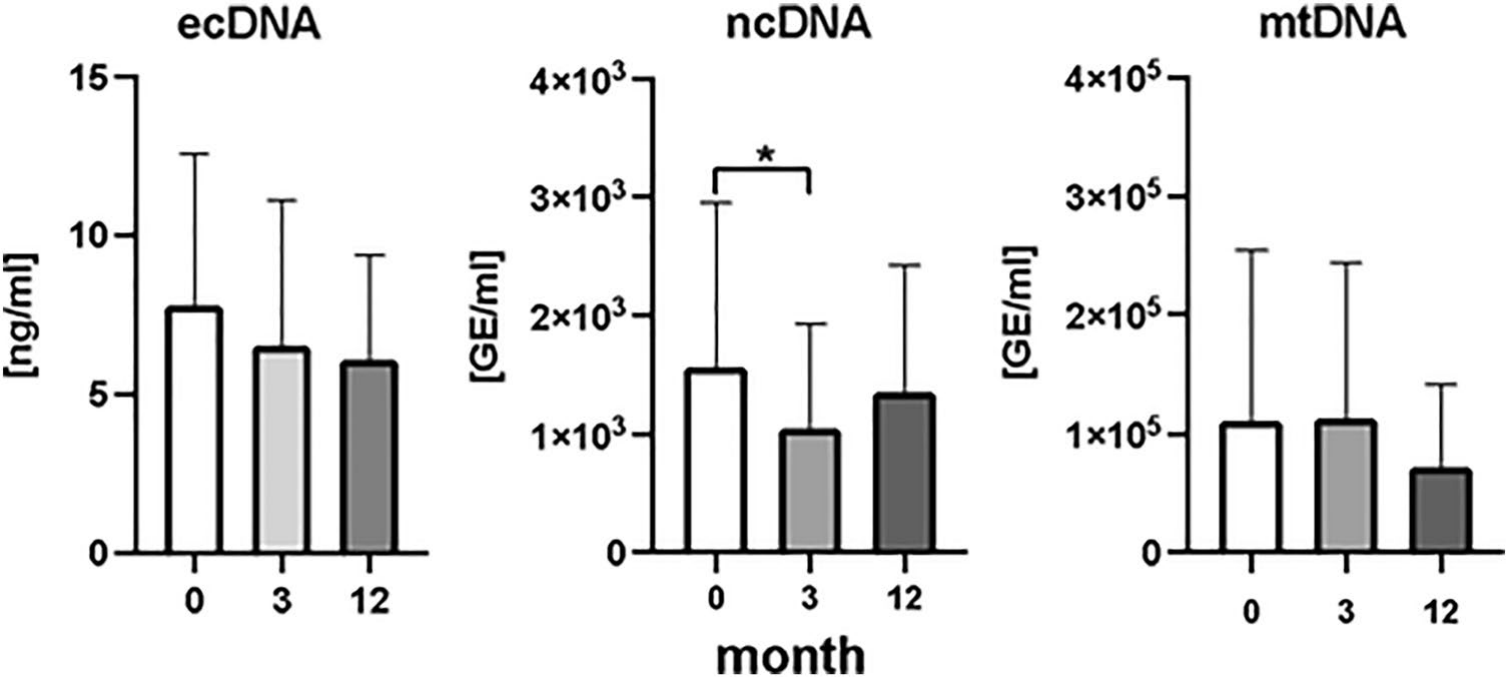
Concentration of total extracellular DNA (ecDNA), nuclear DNA (ncDNA), mitochondrial DNA (mtDNA) in patients with rheumatoid arthritis, before the initiation of bDMARDS and 3 and 12 months during ongoing treatment (*p* < 0,05 = *, *p* < 0.01 = **, *p* < 0.001 = ***).

**Figure 4:**
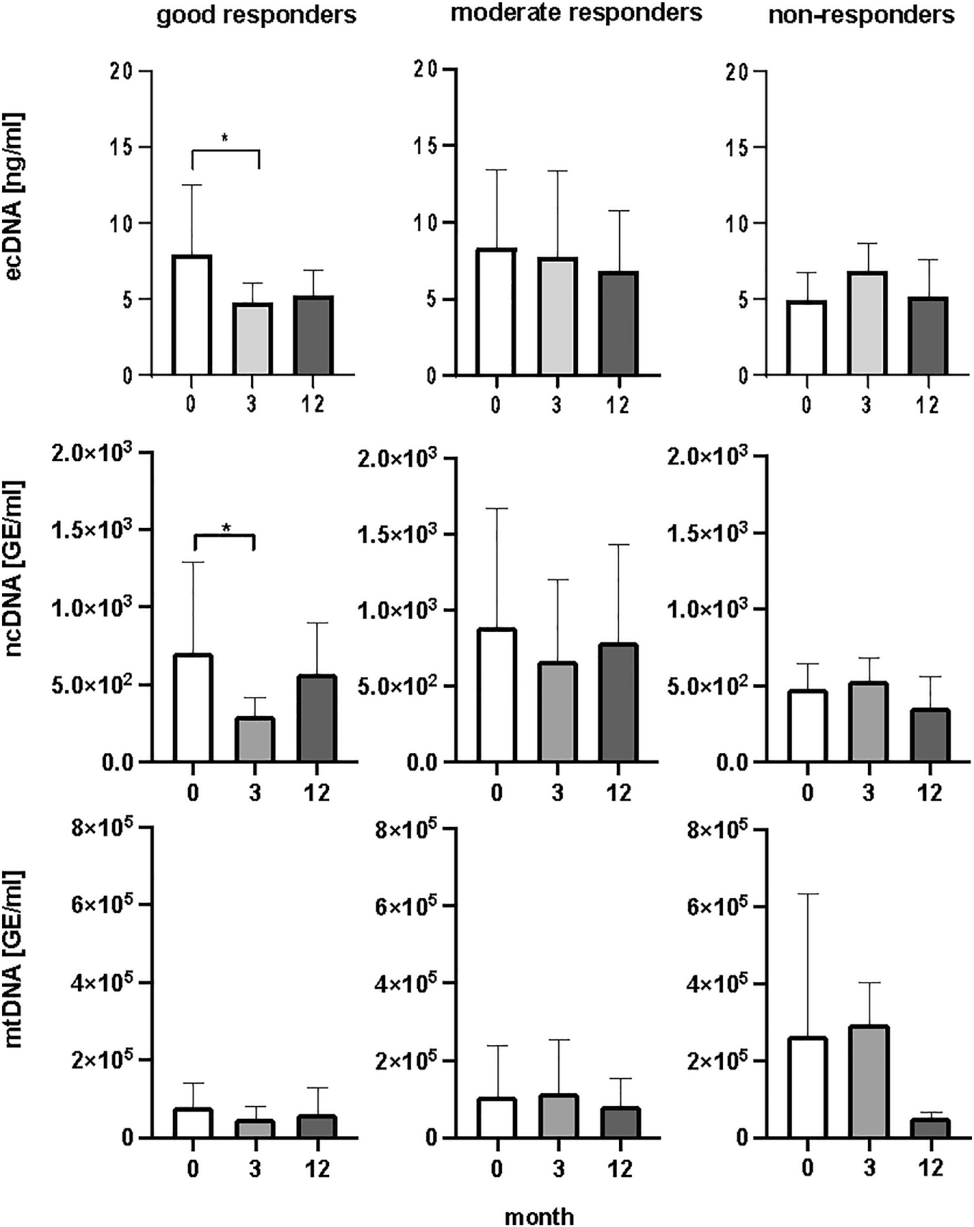
Concentration of total extracellular DNA (ecDNA), nuclear DNA (ncDNA), and mitochondrial (mtDNA), in plasma of patients with rheumatoid arthritis divided according to the treatment response (good, moderate, and non-responders). Before the bDMARDs initiation and 3 and 12 months during ongoing treatment (*p* < 0.05 = *, *p* < 0.01 = **, *p* < 0.001 = ***).

When all patients were analyzed together, dynamics of total plasma ecDNA concentration showed no statistically significant decrease in any of the selected periods of the time (Fig. 3). Statistically significant decrease of ecDNA in plasma was found when RA patients were categorized into response-reflecting groups. In good responders ecDNA significantly decreased by 60%, after the first three months of the biological treatment (Fig. 4), (*p* < 0.05, F = 5.69). Between the third and twelve months after the treatment ecDNA did not show significant decrease. No differences in concentration of ecDNA were found in moderate and non-responders (Fig. 4), (*p* = ns).

Results from real-time PCR, describing the subcellular origin of ecDNA, showed significant change in good responders. Concentration of ncDNA was significantly lower three months after the beginning in good responders (Fig. 4), (*p* < 0.05, F = 6.14). In good responders ncDNA decreased by 58%. In the dynamical changes of mtDNA concentrations no statistical changes were detected. No differences were found between moderate and non-responders in concentration of ncDNA as well as mtDNA before and after bDMARDs treatment (Fig. 4), (*p* = ns).

### Correlation of clinical parameter with ecDNA

**Figure 5:**
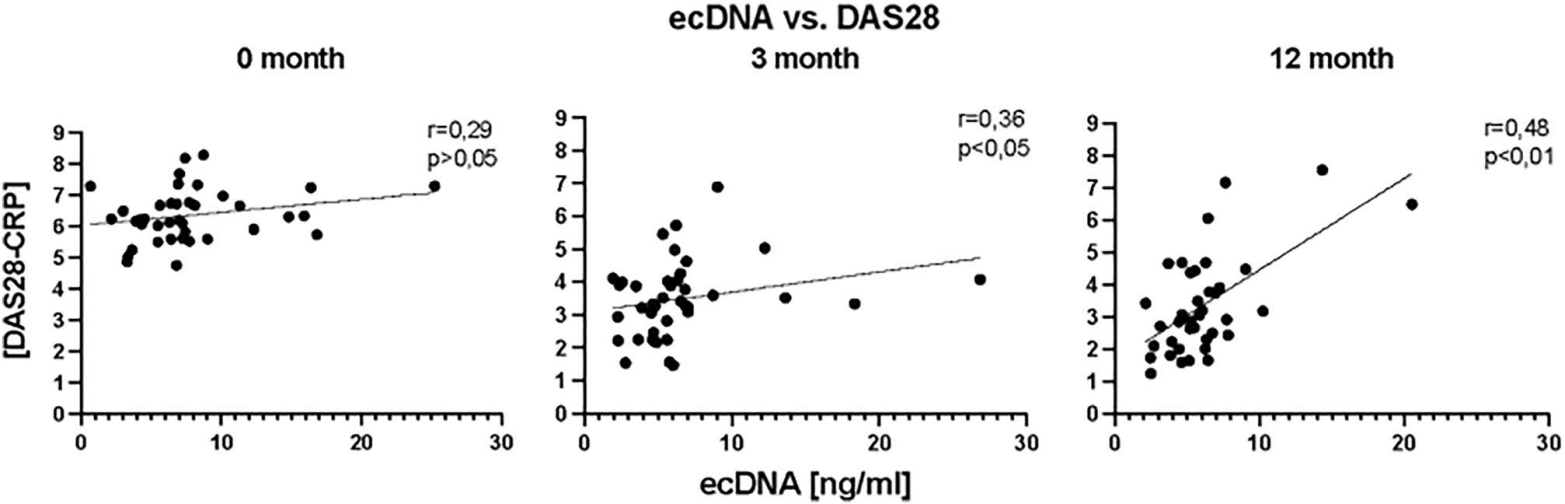
Correlations between concentration of total extracellular DNA (ecDNA) in plasma and disease activity score evaluating 28 joints (DAS28) in specified time points.

**Figure 6:**
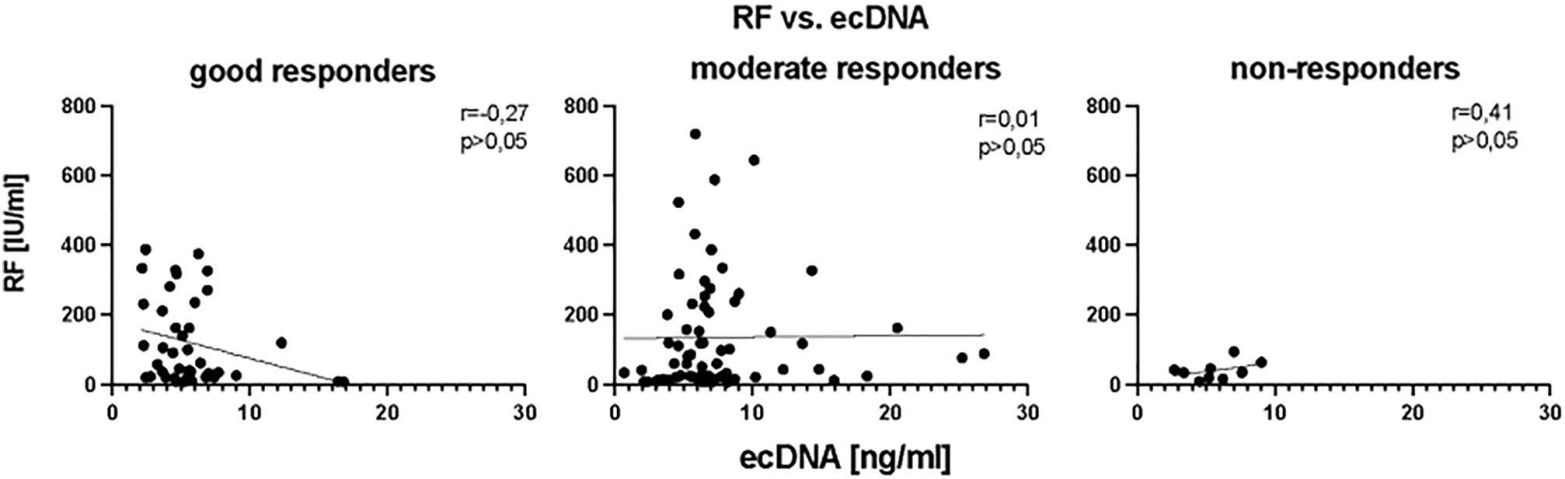
Correlations between concentration of the total baseline extracellular DNA (ecDNA) and rheumatoid factor (RF) in plasma of RA patients according to the treatment response.

**Figure 7:**
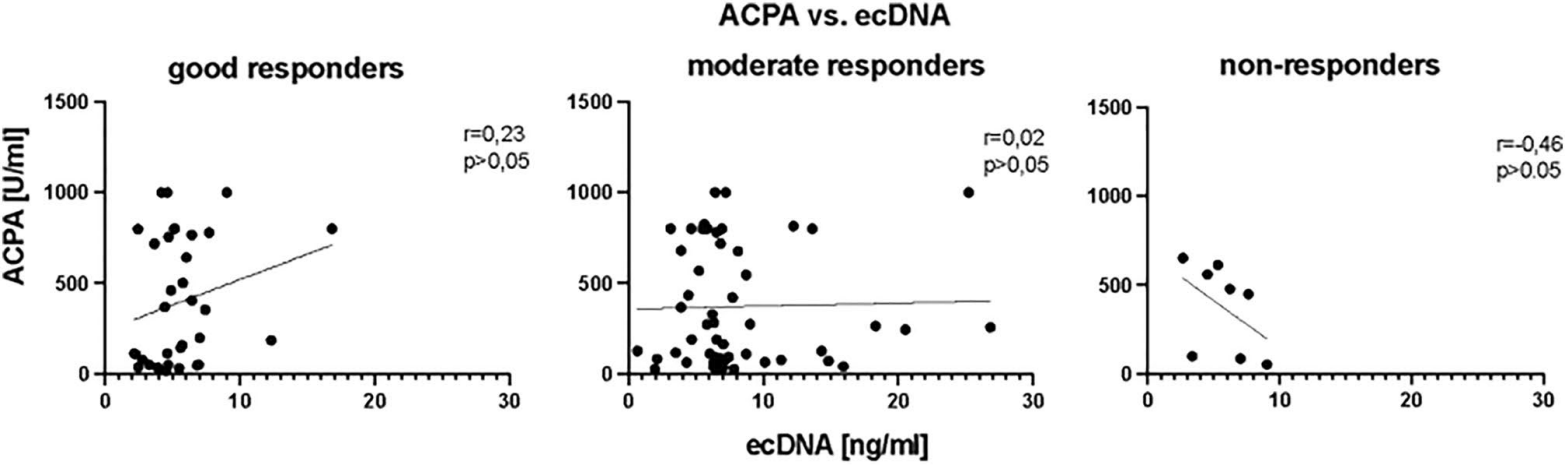
Correlations between concentration of the total baseline extracellular DNA (ecDNA) in plasma and anti-citrullinated protein antibodies (ACPA) of RA patients according to the treatment response.

In the correlation analysis concentration of total ecDNA at the before bDMARDs initiation did not correlate with DAS28 score (Fig. 5), (*p* = ns). However, after three months of bDMARDs application, concentration of total ecDNA in plasma of RA patients positively correlated with DAS28 score (Fig. 5), (r = 0.36, *p* < 0.05, F = 2.3). Positive correlation between these two parameters was also observed after twelve months of treatment (Fig. 5), (r = 0.48, *p* < 0.001, F = 21.50). In the analysis of relation between the rheumatoid factor (RF), anti-citrullinated protein antibodies (ACPA) concentrations regarding the type of the response to the treatment no correlation was detected (Fig. 6, Fig. 7) (*p* = ns).

### DNase activity

**Figure 8:**
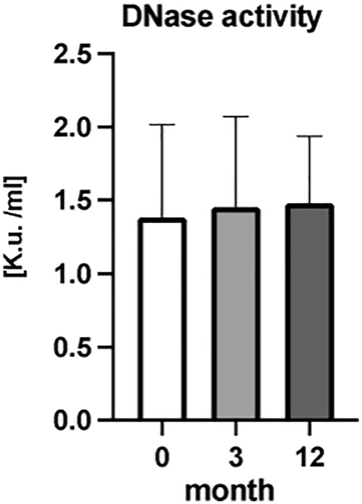
Activity of deoxyribonuclease (DNase) in plasma of rheumatoid patients before the bDMARDs initiation and 3 and 12 months during ongoing treatment (*p* < 0.05 = *, *p* < 0.01 = **, *p* < 0.001 = ***).

**Figure 9:**
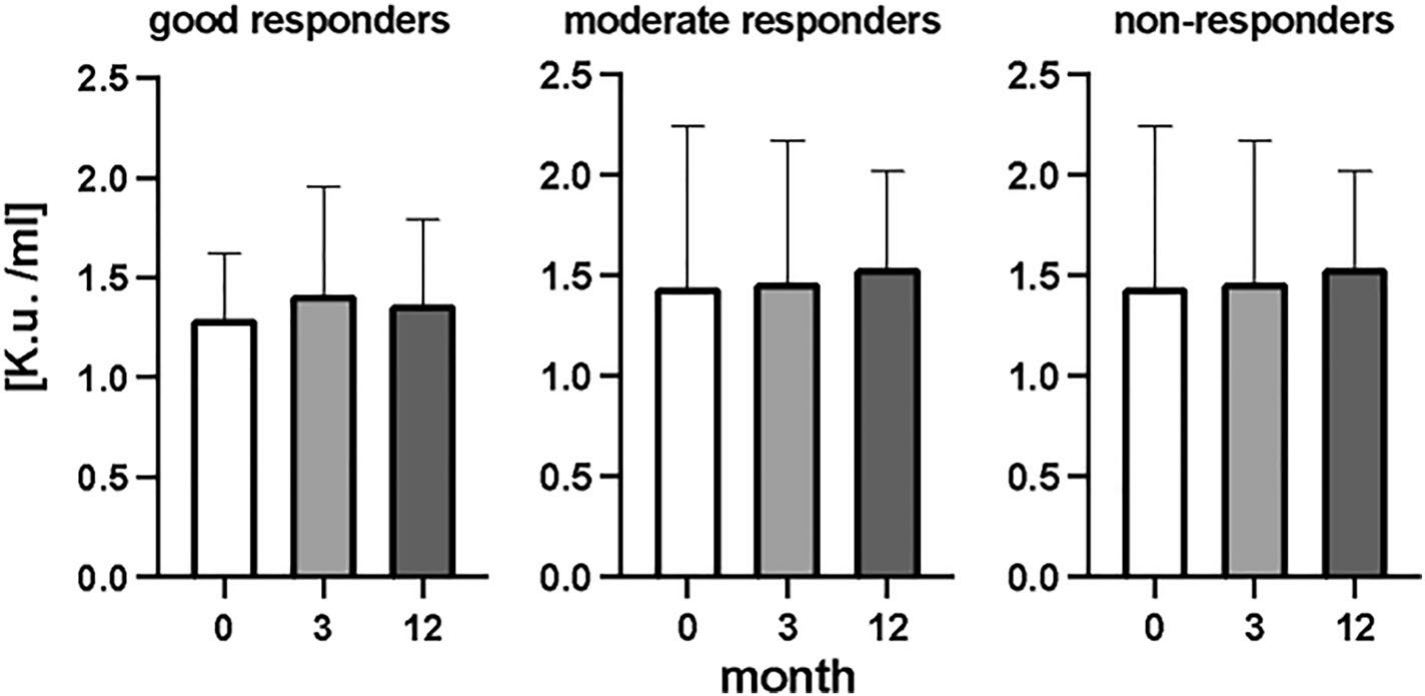
Activity of deoxyribonuclease (DNase) in plasma of patients with rheumatoid arthritis divided into groups based on their response to biological treatment (good, moderate, and non-responders). Before the bDMARDs initiation and 3 and 12 months during ongoing treatment (*p* < 0,05 = *, *p* < 0,01 = **, *p* < 0,001 = ***).

**Figure 10:**
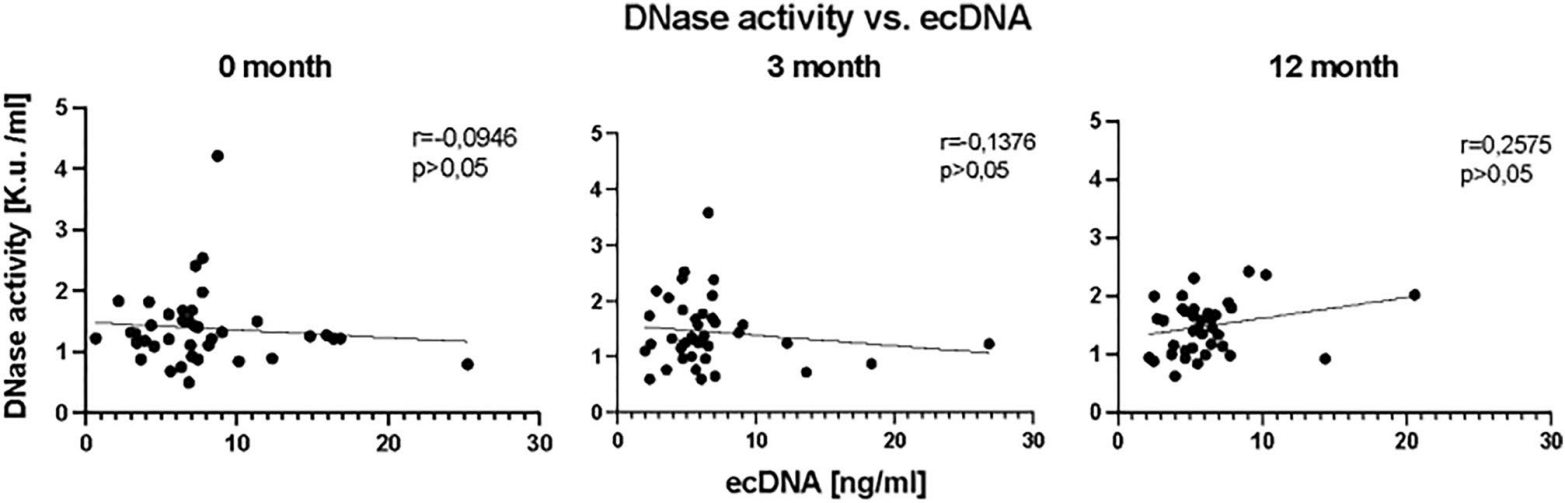
Correlations between DNase activity and ecDNA in plasma in specific time points.

In the determination of DNase activity during the RA, when all patients were analyzed together there were no significant differences in DNase activity (Fig. 8) (*p* = ns). Similarly, no significant changes were observed when RA patients were categorized according to their medical intervention response (Fig. 9) (*p* = ns). Analysis of correlation between concentration of ecDNA and DNase activity in RA patients in three different time points showed no significant correlation (Fig. 10) (*p* = ns).

## Discussion

In the present study we have confirmed the beneficial effects of the bDMARDs on most of the RA patients, since the main clinical status indicators of RA decreased three months after treatment initiation. Decreased values of DAS28 and CRP correlated with the concentration of ecDNA upon 3-month and 12-month bDMARDs administration. Nevertheless, when all patients were analyzed regardless of their treatment response, significant changes were detected neither in total plasma ecDNA nor its subcellular subsets. Statistically significant changes were observed only after categorization of patients regarding their response to treatment. Total ecDNA and ncDNA were decreased only in good responders after three months of bDMARDs initiation. No significant changes were observed in moderate and non-responders to bDMARDs in any time points.

Our previous study^12^ showed that total ecDNA decreased significantly in the plasma of RA patients. We were able to reproduce this finding, but only after exclusion of non-responders and moderate responders. This underlies the importance of patient characteristics for the interpretation of any biomarker study in RA. Another study focused on the exploitability of ecDNA was the study of Hashimoto et al.^11^. In that study ecDNA initially increased during the first 8 weeks, and afterwards concentrations of plasma ecDNA decreased until 12 weeks after the treatment initiation^11^. It is difficult to compare the outcomes since the study design differed, especially in the selection of time points. However, similarly to our results, a significant decrease of ecDNA was observed only in good responders.

In our results, a high variability within the described parameters was shown. The presented variability is predominantly of biological rather than technical origin; for example, the observed relation between DAS28 and ecDNA suggests the importance of more biological factors. Therefore, investigation of factors responsible for biological variability could shed more light on RA disease progression and treatment response in the future.

Activity of DNase, as one of the possible modulators of ecDNA in plasma, was not affected by the treatment in any of the groups. Most studies evaluating DNase activity in various autoimmune diseases reported lower DNase activity^25,26^. However, we cannot prove this for RA as our study design did not include a healthy control group. On the other side the lack of effect of treatment on DNase activity suggests that the observed changes of ecDNA are not a consequence of ecDNA removal, at least not via DNase activity, but rather a consequence of ecDNA production.

In the mentioned studies focusing on ecDNA in RA, the concentration of ecDNA was described as higher than in controls. However, a decreased ecDNA concentration was also reported in RA patients^27^. The basic difference between the studies could be the origin of the sample: in contrast to our study with plasma ecDNA analysis, the mentioned study quantified ecDNA from serum, which is different from plasma. The fact that the sample type might cause fundamentally different results is supported by several studies which describe the increased concentration of ecDNA in plasma from RA patients in comparison with the control group^10,28^. Our study lacks a control group as it was not designed to analyze the effects of RA on ecDNA, but rather the effects of treatment of RA on ecDNA. Our results will, thus, not help to elucidate the association of RA with ecDNA.This study is a follow up to our previously published paper, which showed that bDMARDs treatment decreases ecDNA concentrations in plasma by 26%^12^. A major strength of our studies is the analysis of ncDNA and mtDNA in addition to total ecDNA, as well as the DNase activity that could explain potential differences in ecDNA. In the present study we have also included a wide range of patients with a variable treatment response enabling the identification of variable ecDNA dynamics. Limitations include the relatively infrequent sampling, small number of patients in the non-responder group, but also the unknown tissue origin of the quantified ecDNA. Without identification of its source, ecDNA concentration will remain a nonspecific marker. To better understand the role of ecDNA, in addition to tissues of origin also mechanisms of its release should be analyzed—apoptosis, necrosis, pyroptosis, ferroptosis, but also the production of NETs^28^.

In conclusion, this is the first study describing the dynamics of ecDNA and DNase activity in RA patients before and up to twelve months after initiation of the bDMARDs treatment. This is also a follow up study to our earlier publication in which we described dynamics of ecDNA from the initiation of bDMARDs till six months after. In the presented study we extended the observation time to twelve months. This study shows that application of bDMARDs causes a significant decrease only in good responders with no change of DNase activity in of the evaluated time points. Based on our results we suggest a larger study with more included non-responders with a more frequent sampling. We cannot evaluate if ecDNA is involved in the pathogenesis of RA or it is rather a consequence of the inflammation. However, the fact that anti-inflammatory treatment decreases ecDNA in plasma in good responders supports the hypothesis that ecDNA in RA is mostly of inflammatory origin, likely from immune cells. To describe the role of ecDNA and to confirm if the ecDNA could be used for the monitoring of the treatment responses further studies describing the origin and the mechanism of releasing and clearance should be conducted.

## Methods

### Patients

This study included 39 patients diagnosed with RA according to EULAR 2009 criteria (Table 1). DAS28 (disease activity score evaluating 28 joints), CRP (C-reactive protein), RF (rheumatoid factor), ACPA (anti-citrullinated protein antibodies) were used for clinical patient scoring. In this study, patients were categorized by two approaches. Firstly, patients were analyzed according to the three time points of the bDMARDs treatment, regardless of their treatment response. Time points of the treatment were as follows: before treatment with the bDMARDs started (baseline), three months of treatment, and twelve months of treatment. bDMARDs were administered continuously through the observed period. In the second approach of the analysis, we considered the type of patient response to the treatment. Patients were divided into three different groups: good responders (n = 14), moderate responders (n = 23), and non-responders (n = 3). For this type of categorization, the EULAR response criteria while using the DAS28 were applied. Good responders were categorized with DAS28 ≤ 3.2 at the baseline with subsequent decrease of DAS28 > 1.2. Moderate responders were characterized with range 3.2 < DAS28 ≤ 5.1 and the following decrease of DAS28 > 1.2 or DAS28 > 0.6 and ≤ 1.2. In addition, moderate responders are patients with the baseline DAS28 > 5.1 with posterior decline of DAS28 > 1.2. Non-responders are patients at the baseline DAS28 > 5.1 with the DAS28 > 0.6 and ≤ 1.2 or DAS28 ≤ 0.6. Non-responders are also patients with the baseline DAS28 > 1.2 or 3.2 < DAS28 ≤ 5.1 with the following decrease of DAS28 > 0.6.

**Table 1:**
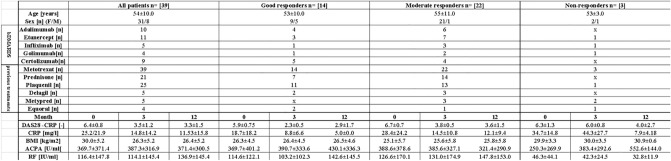
Characteristics of patients with rheumatoid arthritis. Values are presented as mean ± SD. (*CRP *C-reactive protein, *DAS28* disease activity score evaluating 28 joints, *BMI* body mass index, *RF *rheumatoid factor, *ACPA *anti-citrullinated protein antibodies).

The study was approved by the Ethics committee of the National Institute of Rheumatic Diseases in Piešťany. An informed consent was obtained from all participants. All samplings and methods were carried out in accordance with the relevant national and European guidelines and regulations.

### ecDNA analysis

Blood samples (EDTA, Heparin) were collected from RA patients at three above mentioned time points. The samples were centrifuged at 1600 × g for 10 min at 4 °C, in order to separate plasma. Plasma was then stored at − 20 °C until further analyses. For ecDNA analysis, thawed plasma samples were further centrifuged at 16000xg for 10 min at 4 °C. After centrifugation, 200 ul of formed supernatant were used for ecDNA isolation with QIAamp DNA Blood Mini Kit (Qiagen, Hilden, Germany) according to the protocol of the manufacturer. For measuring the concentration of total DNA in plasma from patient samples, Qubit fluorometer and Qubit dsDNA HS Assay Kit (Thermo Fisher, Los Angeles, CA, USA) were used. Technical variability for ecDNA concentration determination is < 5%.

The subcellular origin of plasma ecDNA was described by real-time PCR. For quantification of ncDNA, used primers were based on the detection of human β-globin gene (F:5′-GTGCACCTGACTCCTGAGGAGA-3′, R:5′-CCTTGATACCAACCTGCCCAG-3′). Primers for quantification and detection of mtDNA were based on amplification of part of the human cytochrome B (F: 5´-CCCCACAAACCCCAT TACTAAACCCA-3´, R:5′-TTTCATCATGCGGAGATGTTG GATGG-3′). PCR program was: 1 cycle of 2 min at 95 °C, followed by 45 cycles of 95 °C for 15 s for denaturation. Annealing was set for 1 min at 60 °C. To investigate specificity of the obtained products, the melting curves were analyzed. To obtain final concentrations of ncDNA and mtDNA in genome equivalents (GE/ml), dilutions of known DNA concentrations were performed for standard curve generation. PCR efficiency was calculated for pairs of primers, resulting in 95% and 98% for ncDNA and mtDNA primers, respectively. Technical variability for PCR analysis is < 10%.

### DNase activity

DNase activity was determined from heparin plasma with the single radial enzyme diffusion (SRED) method. 1% agarose gel (20 mM Tris–HCl, pH 7.5, 2 mM MgCl_2_, 2 mM CaCl_2_), with content of DNA isolated from chicken livers (0.035 mg/ml per gel) was needed. For the visualization GoodView Nucleic Acid Stain (SBS Genetech, Beijing, China) was used. For the calibration values the DNase set with RDD buffer (Qiagen, Hilden, Germany) was used. To determine the activity of DNase 1 ul of the DNase stock and of the samples was used. Prepared samples were pipetted into the gel. Gel was incubated overnight at 37 °C in the dark. After the night incubation, the gel was visualized by iBOX (Vision works LP Analysis Software, UVP, Upland, CA, USA). Radial enzyme diffusion was then calculated from the gel by ImageJ software (NIH, Maryland, Bethesda, USA). Activity of DNase was expressed in Kunitz units (K.u.) per ml of heparin plasma^29^. Technical variability for DNase activity is < 10%^30^.

### Statistical analysis

Data obtained in this study were analyzed using software GraphPad Prism 8.1 (La Jolla, California, USA). Based on the Shapiro–Wilk test for normality of distribution, parametric One-way Analysis of Variance (ANOVA) or Friedman’s Test was used for respective parameters. Presented data are shown as mean ± standard deviation. Significant differences were considered when *p* < 0.05.

## Data Availability

The datasets generated in the current study are available from the corresponding author on request.
